# *Uncaria tomentosa* improves insulin sensitivity and inflammation in experimental NAFLD

**DOI:** 10.1038/s41598-018-29044-y

**Published:** 2018-07-20

**Authors:** Layanne C. C. Araujo, Karla B. Feitosa, Gilson M. Murata, Isadora C. Furigo, Simone A. Teixeira, Camila F. Lucena, Luciene M. Ribeiro, Marcelo N. Muscará, Soraia K. P. Costa, José Donato, Silvana Bordin, Rui Curi, Carla R. O. Carvalho

**Affiliations:** 1 Department of Physiology and Biophysics, University of São Paulo, São Paulo, 05508-900 Brazil; 2 Department of Pharmacology, Institute of Biological Science, University of São Paulo, São Paulo, 05508-900 Brazil; 3 Interdisciplinar Post-Graduate Program in Health Sciences, Cruzeiro do Sul University, São Paulo, SP Brazil

## Abstract

We investigated the effect of the crude herbal extract from *Uncaria tomentosa* (UT) on non-alcoholic fatty liver disease (NAFLD) in two models of obesity: high fat diet (HFD) and genetically obese (ob/ob) mice. Both obese mouse models were insulin resistant and exhibited an abundance of lipid droplets in the hepatocytes and inflammatory cell infiltration in the liver, while only the HFD group had collagen deposition in the perivascular space of the liver. UT treatment significantly reduced liver steatosis and inflammation in both obese mouse models. Furthermore, serine phosphorylation of IRS-1 was reduced by 25% in the HFD mice treated with UT. Overall, UT treated animals exhibited higher insulin sensitivity as compared to vehicle administration. In conclusion, *Uncaria tomentosa* extract improved glucose homeostasis and reverted NAFLD to a benign hepatic steatosis condition and these effects were associated with the attenuation of liver inflammation in obese mice.

## Introduction

Non-alcoholic fatty liver disease (NAFLD) is characterized by ectopic fat accumulation in the liver of people with no previous history of alcohol abuse, and affects about 1 billion people worldwide. NAFLD is a global public health problem associated with an increased prevalence of obesity, and is regarded as a hepatic manifestation of the metabolic syndrome^1^. NAFLD has a prevalence of approximately 30% in the adult population of developed countries, and it has also become a frequent liver disease in children, due to increased prevalence of childhood obesity^2^. NAFLD can be classified as simple steatosis or nonalcoholic steatohepatitis (NASH)^3–5^. Despite the benign clinical course of steatosis, one third of people with NAFLD have NASH. NASH, on other hand, is a progressive disease that can progress to liver cirrhosis and hepatocellular carcinoma^6^.

Despite some tremendous worldwide efforts to combat obesity, the synthetic drugs approved by the Food and Drug Administration (FDA) such as: orlistat, lorcaserin, fentermin-topiramate, rimonabant, and sibrutamin/meridian have collateral side effects that have made some of them inappropriate for prolonged usage. In fact, the use of these drugs has been associated with an increased occurrence of hypertension, cardiac diseases, hepatic failure, as well as cognitive and psychiatric effects. Surgical interventions, on the other hand, are associated with co-morbidities and have high costs. Thus, our goal is to investigate a novel, prospective therapeutic option against obesity and its co-morbidities, based on the crude extract of *Uncaria tomentosa*.

The crude extract of *Uncaria tomentosa*, also known as cat’s claw, contains a mixture of quinovic acid glycosides, polyphenols and tetracyclic or pentacyclic oxindolic alkaloids, such as pteropodine, isopteropodine, speciophylline, uncarine F, mitraphylline and isomitraphylline. These secondary metabolites have anti-inflammatory properties^7–11^. In parallel with its known anti-inflammatory effect, this herbal medicine, has been used to reduce adverse chemotherapy side-effects, gastric ulcer, viral infections, urinary tract disease, arthritis and general inflammatory conditions^12,13^.

Here we administered an equivalent human dosage of *Uncaria tomentosa* (UT) for five consecutive days to two distinct rodent models of obesity: high fat diet (HFD) and genetically obese (ob/ob) mice. The results demonstrated that obese mice administered UT showed an improvement in insulin sensitivity and NASH, as well as decreased lipid accumulation and inflammation. Our data suggests that the herbal medicine, cat’s claw, has the potential to combat insulin resistance and NAFLD, which are two features associated with obesity.

## Results

### Uncaria tomentosa reduces BMI and increases energy expenditure in obese mice

In order to investigate the effect of the herbal extract on caloric intake, body weight, whole-body energy metabolism, and respiratory exchange ratio (RER), mice fed either a standard diet (SD) or high fat diet (HFD) and ob/ob mice (genetically obese due to hypoleptinemia) were submitted to CLAMS analysis.

**Figure 1:**
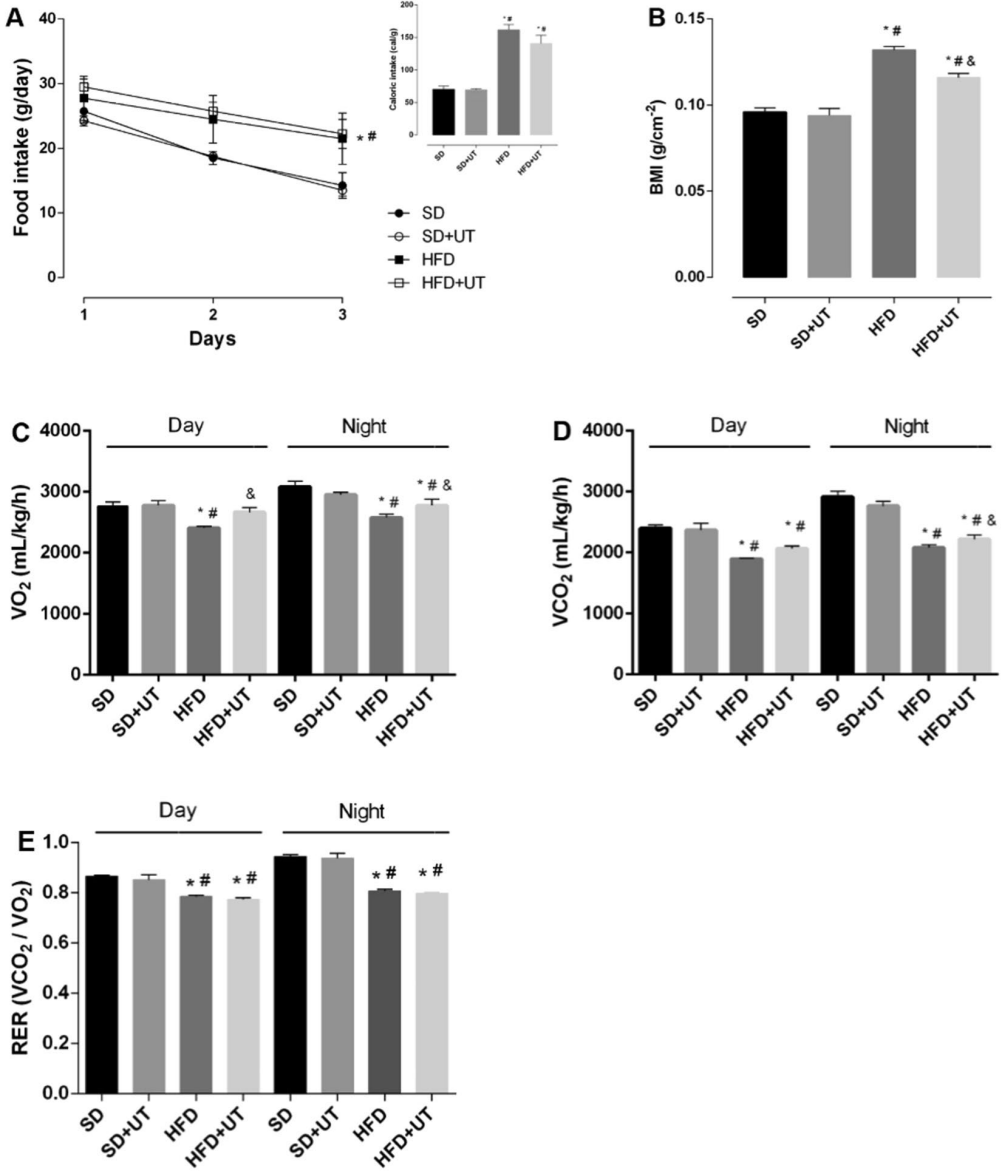
Effect of *Uncaria tomentosa* (UT) on the metabolic parameters of HFD animals. Adult male C57BL/6 mice received either standard chow, representing the control group (SD), or high-fat diet (HFD) for a 12-week period. Seven days before the end of the feeding regimen period, the animals were isolated in the CLAMS chambers. After two days of adaptation, the mice received either vehicle (saline) or UT, 50 mg.kg^−1^ b.w., once a day for 5 days (SD+UT and HFD+UT), via gavage. During the last 3 days, food intake to calculate the caloric intake (**A**), O_2_ consumption (VO_2_) (**C**), CO_2_ production (VCO_2_) (**D**), and the respiratory coefficient (RER) (**E**) were measured. Body mass index was calculated at the end of the period (**B**). The results are represented as mean ± SEM, n = 4 to 5. The statistical differences as indicated by two-way ANOVA were as follows: *p < 0.05 (*vs*. SD); ^#^p < 0.05 (*vs*. SD+UT); ^&^p < 0.05 (*vs*. HFD).

The HFD and HFD+UT groups had an increase in the food and calorie intake, as compared to the SD group, and the UT treatment had no impact on these parameters (Fig. 1A). On the other hand, the observed 44% increase in the body mass index (BMI) of the HFD group, when compared with the SD group, was reduced to 23% in the HFD+UT group (*p* < 0.05) (Fig. 1B). The HFD mice also displayed a lower energy expenditure in both the dark and light periods, as indicated by the reduction of VO_2_ to 83% (dark) and 78% (light) (Fig. 1C), VCO_2_ to 71% (dark) and 65% (light) (Fig. 1D), and RER to 85% (dark) and 83% (light) compared with the SD group (dark) at 100% (Fig. 1E) (p < 0.05). The HFD+UT mice had ~39% and ~72% reversion/protection of VO_2_ and ~16% and ~38% reversion/protection of VCO_2_ in the nighttime and daytime periods, respectively (*p* < 0.05) (Fig. 1C,D). The phytochemical had no impact on the RER (Fig. 1E), nor in the 8-fold increase of blood leptin levels in HFD mice, when compared with lean SD mice (data not shown).

**Figure 2:**
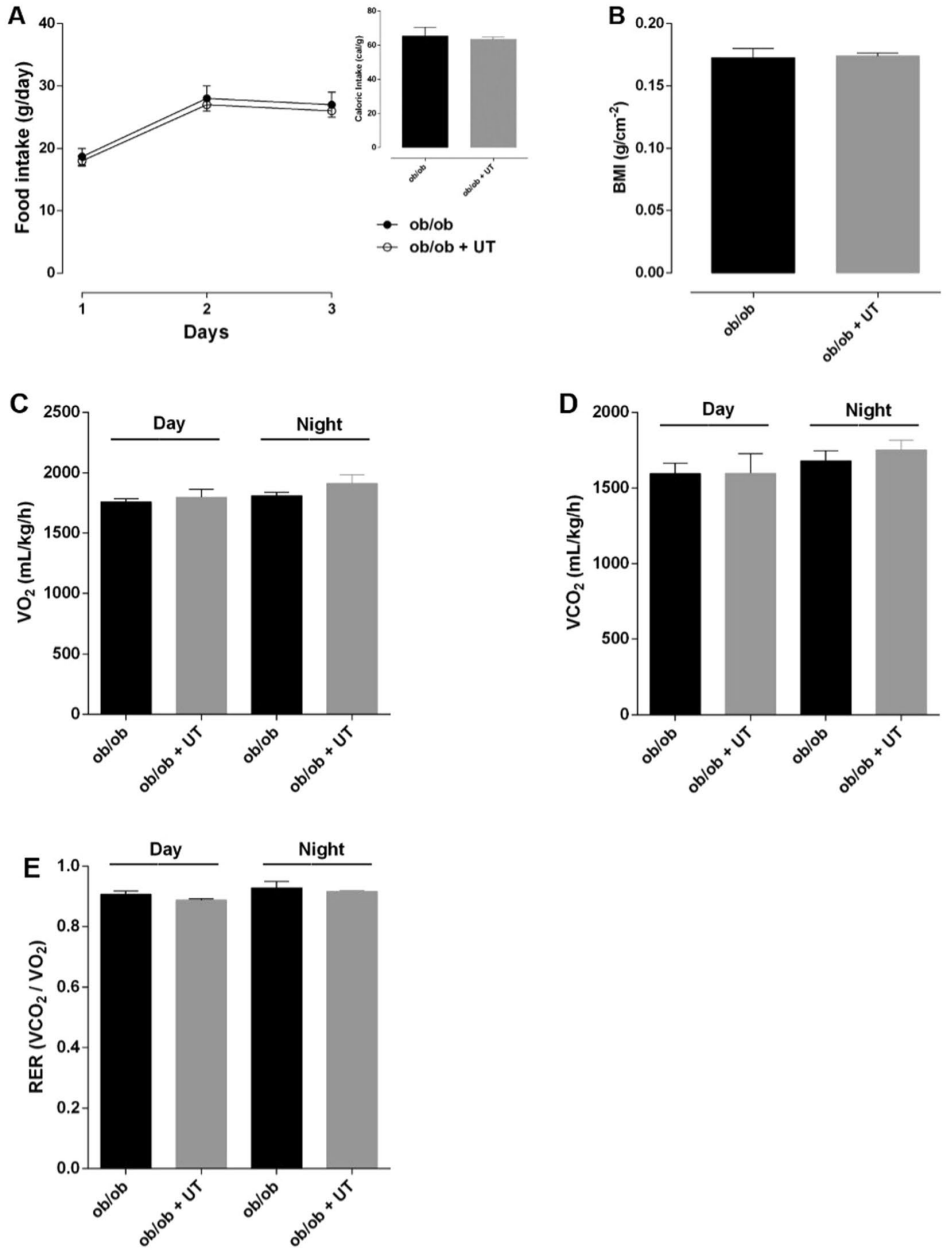
Effect of *Uncaria tomentosa* (UT) on metabolic parameters of ob/ob animals. Adult ob/ob mice fed with standard chow were divided into two groups: control (ob/ob) and UT (ob/ob+UT), which received 50 mg.kg^−1^ b.w. of crude plant extract (UT) diluted with saline, for 5 consecutive days, once a day, via gavage. The animals were isolated in CLAMS chambers. After two days of adaptation, the mice received either vehicle (saline) or UT, 50 mg.kg^−1^ b.w., once a day for 5 days (ob/ob+UT), via gavage. During the last 3 days, food intake to calculate the caloric intake (**A**), O_2_ consumption (VO_2_) (**C**), CO_2_ production (VCO_2_) (**D**), and the respiratory coefficient (RER) (**E**) were measured. Body mass index was calculated at the end of the period (**B**). The results are represented as mean ± SEM, n = 4 to 5. The statistical difference was analyzed by Student’s *t*-test or two-way ANOVA.

In the ob/ob mice, the food and calorie intake (Fig. 2A), the BMI (Fig. 2B), and the energy expenditure parameters (Fig. 2C–E) were similar between the vehicle and UT treated mice.

These observation may result from the lack of leptin in ob/ob mice while, in HFD mice, the ameliorating effects of UT would associate with a decrease in HFD-induced hypothalamic leptin-resistance, rather than a decrease in leptin circulating levels.

### Uncaria tomentosa reduces fasting blood glucose levels, improves blood glucose homeostasis, and improves liver insulin signaling in obese mice

**Figure 3:**
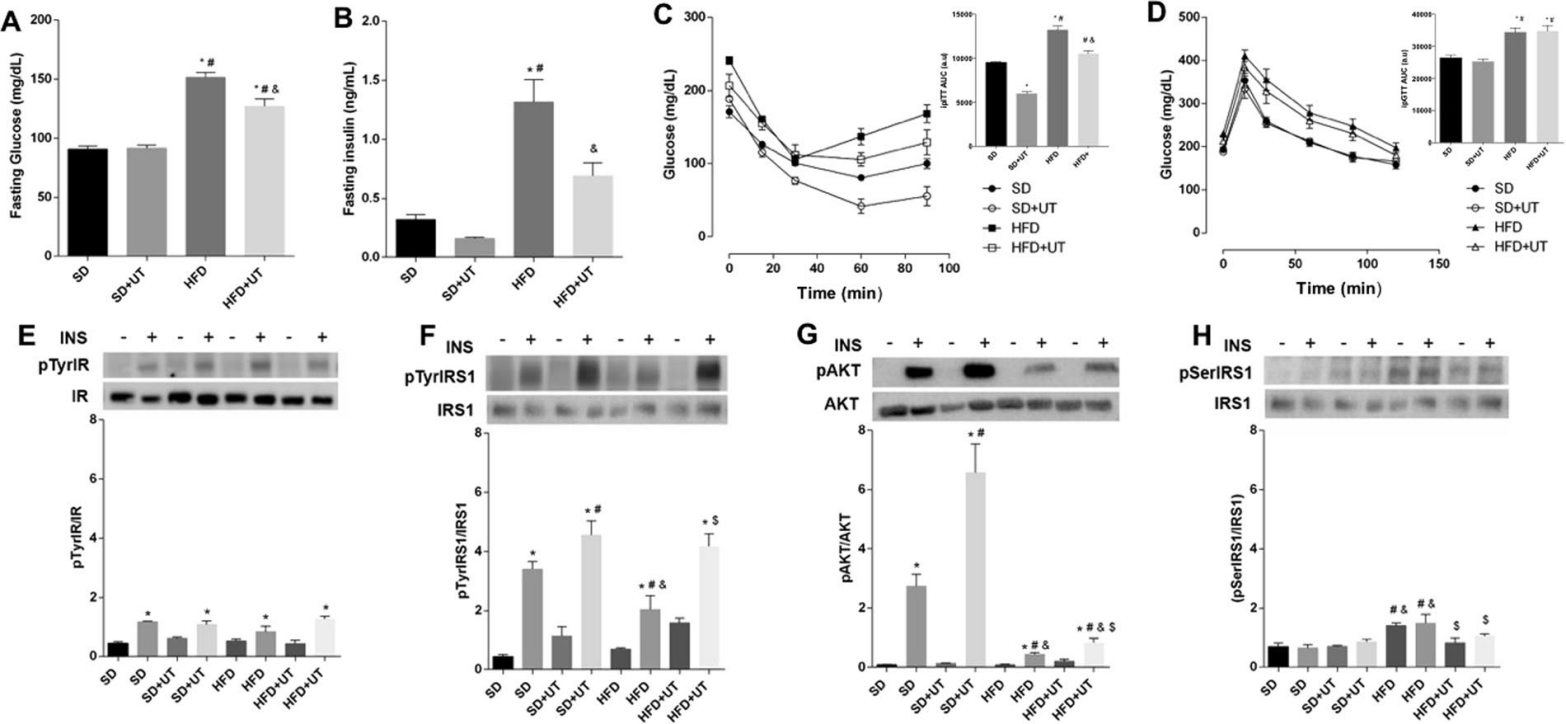
Effect of *Uncaria tomentosa* (UT) on glucose metabolism of HFD mice. The animals were fed a HFD for 12-week period and then administered 50 mg.kg^-1^ b.w. of crude plant extract (UT), once a day, for 5 consecutive days or saline under the same regimen. The groups were: standard diet (SD), standard diet+UT (SD+UT), high fat diet (HFD) and high fat diet+UT (HFD+UT). The results are presented as mean ± SEM. The statistical differences as indicated by two-way ANOVA. Fasting blood glucose is represented in **(A**); *p < 0.0001 (SD *vs*. HFD, SD *vs*. HFD+UT), ^#^*p* < 0.0001 (SD+UT *vs*. HFD, SD+UT *vs*. HFD+UT), ^&^*p* < 0.0001 (HFD *vs*. HFD+UT); n = 5. Fasting blood insulin concentration (**B**); *p < 0.01 (SD *vs*. HFD), ^#^*p* < 0.01 (SD+UT *vs*. HFD), ^&^*p* < 0.05 (HFD *vs*. HFD+UT), n = 4. GTT is represented in (**C**); *p < 0.05 (SD *vs*. HFD; SD *vs*. HFD+UT), ^#^*p* < 0.05 (SD+UT *vs*. HFD; SD+UT *vs*. HFD+UT; n = 10. Insulin tolerance test (ITT) is represented in (**D**); *p < 0.05 (compared to SD), ^#^*p* < 0.05 (compared to SD+UT), ^&^*p* < 0.05 (compared to HFD), n = 5. Phosphorylated proteins (**E**–**H**) were represented by the ratio of the optical densitometry of phosphorylated protein and total protein expression. The statistical differences as indicated by two-way ANOVA were as follows: *p < 0.05 (stimulated groups (+) *vs*. not stimulated groups (−) with insulin), ^#^*p* < 0.05 (compared to SD+), ^&^*p* < 0.05 (compared to SD+UT), ^$^*p* < 0.05 (compared to HFD+); n = 6.

As expected, the HFD group presented impaired glucose homeostasis with increased fasting blood glucose levels (151 ± 4 *vs*. 90 ± 2 mg/dL, *p* < 0.05) (Fig. 3A), elevated insulin levels (1.3 ± 0.2 *vs*. 0.3 ± 0.04 ng/mL, *p* < 0.05) (Fig. 3B), reduced insulin sensitivity (AUC: 9538 ± 440 *vs*. 13223 ± 449), p < 0.05) (Fig. 3C) and impaired glucose tolerance (AUC: 26361 ± 875.2 *vs*. 34354 ± 1358, *p* < *0.05*)(Fig. 3D). The HFD+UT group showed a 15% reduction in the fasting blood glucose levels (127 ± 6 *vs*. 151 ± 4 m/dL, p < 0.05) (Fig. 3A) and a 45% reduction in the insulin blood level concentration (0.7 ± 0.1 *vs*. 1.3 ± 0.2 ng/mL, p < 0.05) (Fig. 3B). While the treatment improved insulin sensitivity, when compared to HFD mice (Fig. 3C), there was no observable impact on glucose tolerance (Fig. 3D).

Comparisons of the IR tyrosine phosphorylation status indicated that there was no difference among the groups, however, increased IR tyrosine phosphorylation was observed following acute insulin stimulation, as indicated by the plus signal (Fig. 3E). On the other hand, the HFD group exhibited a 41% (p < 0.05) reduction in insulin induced IRS-1 tyrosine phosphorylation (Fig. 3F) and an 85% (p < 0.05) reduction in AKT phosphorylation (Fig. 3G), as compared to the SD group. The UT treatment induced an augmentation in the insulin induced phosphorylation of IRS-1 in the SD+UT group and HFD+UT group by 10% (p < 0.05) and 40% (p < 0.05), respectively. Additionally, UT treatment improved insulin induced AKT phosphorylation by nearly two-fold in the SD+UT group (p < 0.05) and by 15% (p < 0.05) in the HFD+UT group (Fig. 3F,G).

Furthermore, the IRS-1 Ser^307^ phosphorylation was increased in the HFD group, when compared to the SD group (200% *vs*. 100%, respectively, *p* < 0.05), and this phosphorylation event was reduced to 143% (*p* < 0.05) in the HFD+UT group (Fig. 3H).

**Figure 4:**
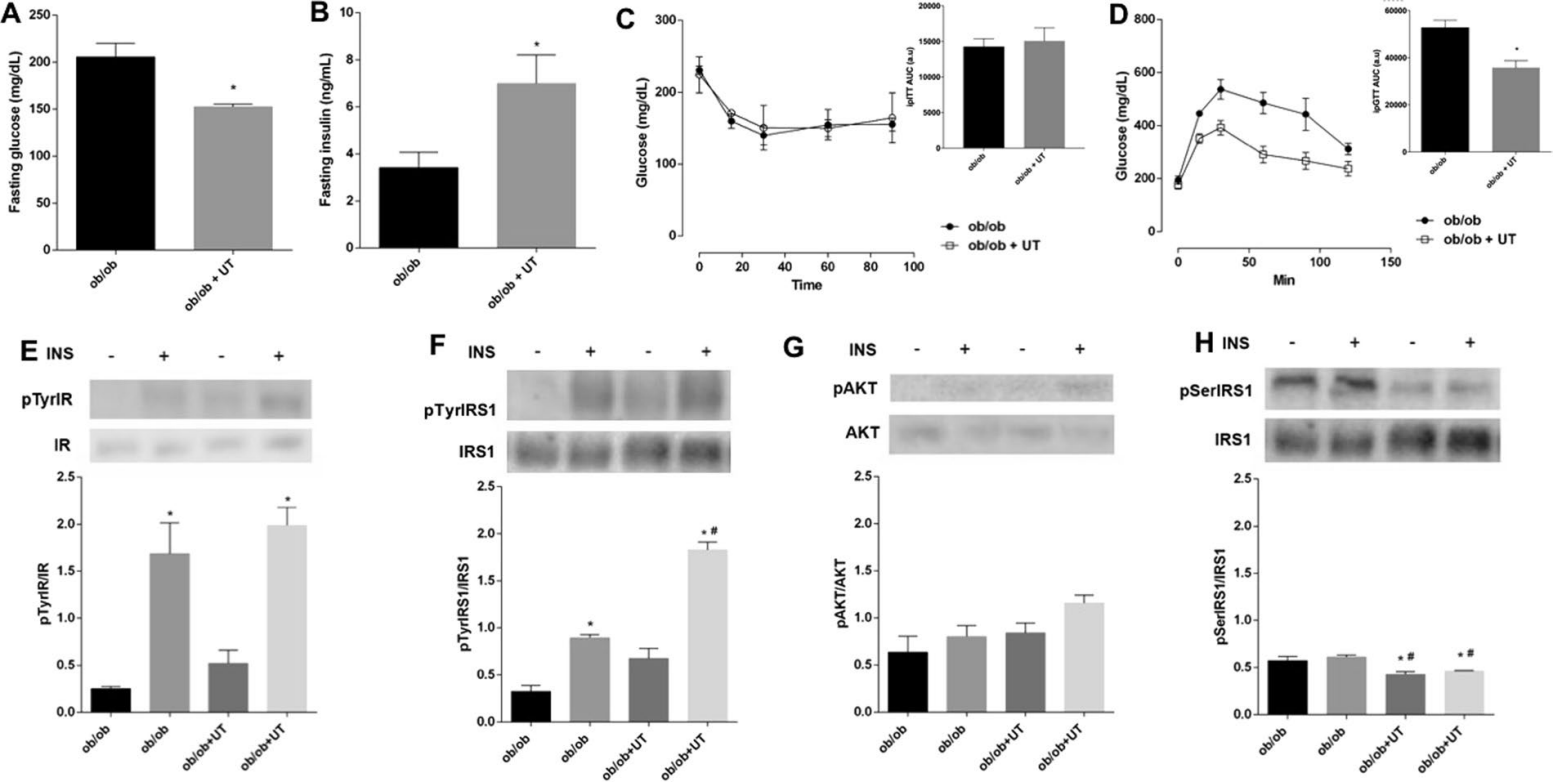
Effect of *Uncaria tomentosa* (UT) on glucose metabolism of ob/ob mice. The adult male mice were fed with standard chow and then administered 50 mg.kg^−1^ b.w. of crude UT extract, once a day, for 5 consecutive days or saline under the same regimen. The results are presented as mean ± SEM. The statistical differences as indicated by Student’s *t*-test. Fasting blood glucose is represented in (**A**) n = 6, p < 0.0001. Fasting blood insulin concentration (**B**); *p* < 0.05, n = 4–7. GTT is represented in (**C**) n = 6, p < 0.01. ITT is represented in (**D**), n = 5. Phosphorylated proteins (**E**–**H**) were represented by the ratio of the optical densitometry of phosphorylated protein and total protein expression, the statistical differences as indicated by two-way ANOVA were as follows: *p < 0.05 (4E and 4F: stimulated groups (+) *vs*. not stimulated groups (−) with insulin), *p < 0.05 (4 H: ob/ob- *vs*. ob/ob+UT−; ob/ob- *vs*. ob/ob+UT+), ^#^*p* < 0.05 (ob/ob+ *vs*. ob/ob+UT−, obo/b+ *vs*. ob/ob+UT+), n = 5.

The ob/ob+UT group exhibited a 20% reduction in the fasting blood glucose levels (163 ± 11 *vs*. 205 ± 15 mg/dL, *p* < 0.05) (Fig. 4A) associated to increased insulin blood level (7.0 ± 1.2 *vs*. 3.4 ± 0.7 ng/mL, p < 0.05) (Fig. 4B). The ITT was similar between the treated and untreated ob/ob groups (Fig. 4C), although the UT treatment improved glucose tolerance (AUC: 35730 ± 2969 *vs*. 52759 ± 3120, p < 0.05) (Fig. 4D).

The ob/ob mice also exhibited increased IR tyrosine phosphorylation after insulin infusion, and the extents were similar with or without the UT treatment (Fig. 4E). On the other hand, the IRS-1 tyrosine phosphorylation after insulin stimulation was two-fold higher (p < 0.05) in the ob/ob+UT group as compared to the saline vehicle control (Fig. 4F). It was also observed that in the ob/ob mice insulin infusion or UT treatment had no influence on the degree of AKT phosphorylation (Fig. 4G). Furthermore, the serine phosphorylation of IRS-1 was reduced by 20% (p < 0.05) in the ob/ob+UT group (Fig. 4H).

In summary, there appears to be an impairment in the insulin signaling pathway in the liver via a reduced insulin induced IRS-1 tyrosine phosphorylation. This effect was associated with an enhanced IRS-1 phosphorylation at Ser^307^, and UT treatment reversed the degree of phosphorylation.

### Uncaria tomentosa reduces fat liver disease related to obesity

**Figure 5:**
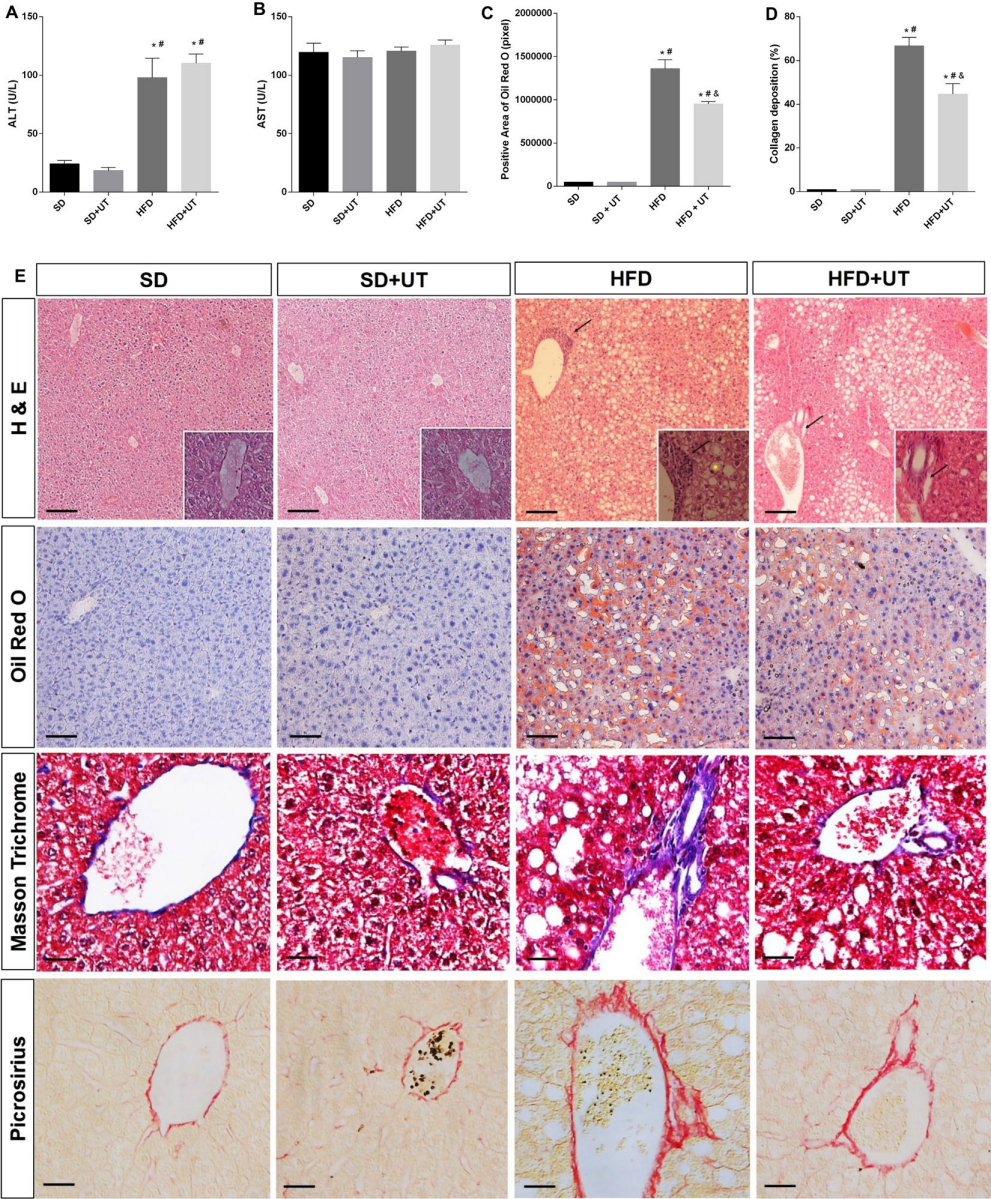
Hepatic enzyme activities are represented in (**A,B**), lipid droplets in (**C**), and collagen fibril deposition in (**D**). Morphology of liver (**E**) in Hematoxylin/Eosin staining the lipid droplets with ballooning hepatocytes (yellow *) and inflammatory cellular infiltrate (black arrow) in the perivenular areas of the liver, and in Oil Red O staining the fat accumulation is shown in red. Black bar represents a scale of 50 µm, objective 20×. In Masson trichrome and Picrosirius staining the collagen fibrils, in the perivenular area, are in blue and red, respectively, the black bar represents a scale of 100 µm objective 40×. The results are represented as mean ± SEM. The statistical differences as indicated by two-way ANOVA were as follows: *p < 0.0001 (SD vs. HFD, SD *vs*. HFD+UT), ^#^*p* < 0.0001 (SD+UT *vs*. HFD; SD+UT *vs*. HFD+UT), ^&^*p* < 0.001 (HFD *vs*. HFD+UT); n = 4.

Plasma enzymes aspartate transaminase (ALT) and alanine transaminase (ALT) activities were measured in order to evaluate the extension of the eventual liver lesion. The HFD induced an increase of ALT activity as compared to the SD group (98 ± 3 *vs*. 24 ± 16 U/L, *p* < 0.05) and the UT treatment had no significant effect on this activity (110 ± 8 U/L) (Fig. 5A). The activity of AST remained unchanged, regardless of diet or UT treatment (Fig. 5B).

In order to differentiate between different types NAFLD (i.e. NAFL *vs*. NASH), we performed histological analysis using hematoxylin/eosin (HE), Oil Red O (ORO), Masson Trichrome and Picrosirius stains. We considered hepatic steatosis when the intracellular fat accumulation was observed in more than 5% of the hepatocytes. NAFL corresponded to the presence of steatosis associated with ballooning but with no inflammatory infiltrate, or with no ballooning but mild inflammatory infiltrate. NASH classification required the association with steatosis, hepatocyte ballooning, lymphocytic and neutrophilic inflammatory infiltrate and higher collagen deposits in the perivenular areas of the liver^14^.

The abundance of neutral intracellular lipids was quantified by color intensity using the ORO staining method. There were no detectable neutral intracellular lipids in the liver slices from the SD group. The HFD+UT mice displayed a 30% reduction in intracellular fat, when compared to livers of the HFD group (*p* < 0.05) (Fig. 5C). Furthermore, in the HFD livers, there was a 70% increase in the amount of detectable collagen deposition in the perivenular spaces, and the HFD+UT group showed a 45% improvement in this parameter (Fig. 5D).

Livers of the lean SD group had no detectable vesicles in the hepatocytes stained with HE, while the HFD mice displayed macrovesicular steatosis in 28% of the counted hepatocytes and balloon shaped. There was also mononuclear cell inflammatory infiltrate present in the perivenular space of the liver, accompanied by clearly identified collagen fibrils in the Masson trichrome and Picrosirius stained samples (Fig. 5E).

**Figure 6:**
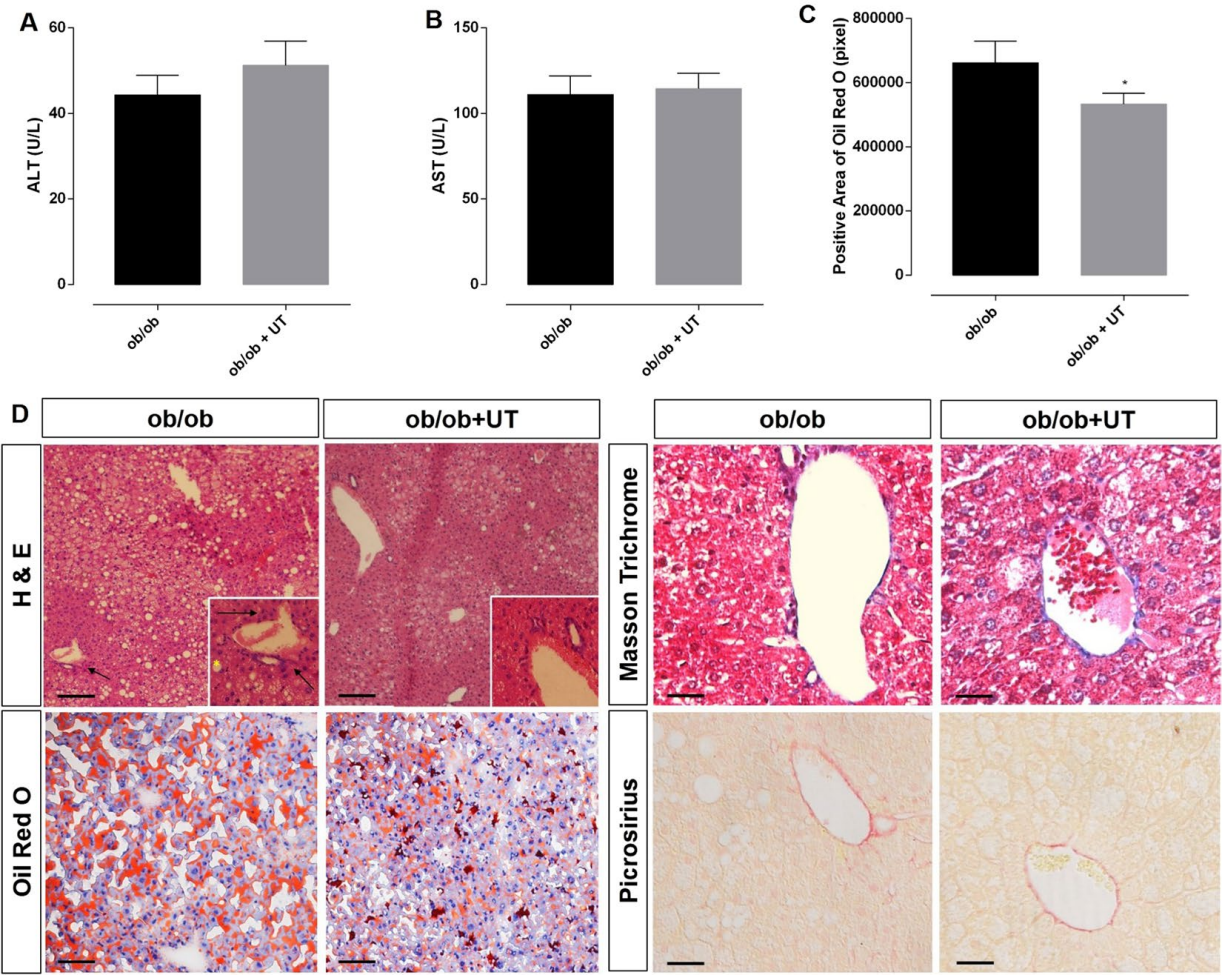
Hepatic enzyme activities are represented in (**A,B**), lipid droplets in (**C**). Morphology of liver (**D**) in Hematoxylin/Eosin staining the lipid droplets with ballooning hepatocytes (yellow*) and inflammatory cellular infiltrate (black arrow) in the liver perivenular areas, and in Oil Red O staining the fat accumulation is shown in red. Black bar represents a scale of 50 µm, objective 20×. In Masson trichrome and Picrosirius staining the collagen fibrils, in the perivenular area, are in blue and in red, respectively, the black bar represents a scale of 100 µm objective 40×. The ob/ob animals showed no collagen deposition. The results are presented as mean ± SEM. The statistical difference as indicated by Student’s *t*-test was: *p < 0.05, n = 4.

UT treatment had no effect on the activities of ALT and AST in the ob/ob group (Fig. 6A,B, respectively).

The ob/ob+UT group displayed a 20% reduction in the hepatocyte lipid content (80% *vs*.100%, *p* < 0.05) (Fig. 6C). The lipid droplets within the hepatocytes from the ob/ob mice were mainly microvesicular, but with some ballooning, and essentially no inflammatory infiltrate was identified in the perivenular areas of the liver. These features were associated with a low amount of collagen staining in the liver perivenular spaces, similar to that observed in the lean control mice (Fig. 6D).

Using a classical tissue staining procedure we were able to characterize the type of NAFLD: (1) NAFL, which is the benign form, exhibits hepatic steatosis and balloon shaped hepatocytes, and (2) NASH exhibits hepatic steatosis, balloon shaped hepatocytes, inflammatory cellular infiltrate and enhanced collagen deposits in the perivascular space. Based on our results the UT treatment was able to reverse NASH pattern to the less harmful NAFL phenotype.

### Uncaria tomentosa reduces liver inflammatory state

The inflammatory state of the NAFLD was evaluated through: (1) western blot analysis of the TNF-α/JNK/Ikkβ/NFkB intracellular pathway, (2) the genetic expression of IL-1β, IL-10, F4-80 and arginase-1, and (3) the immunohistochemistry with an anti-F4-80 antibody in the liver of the obese mice.

**Figure 7:**
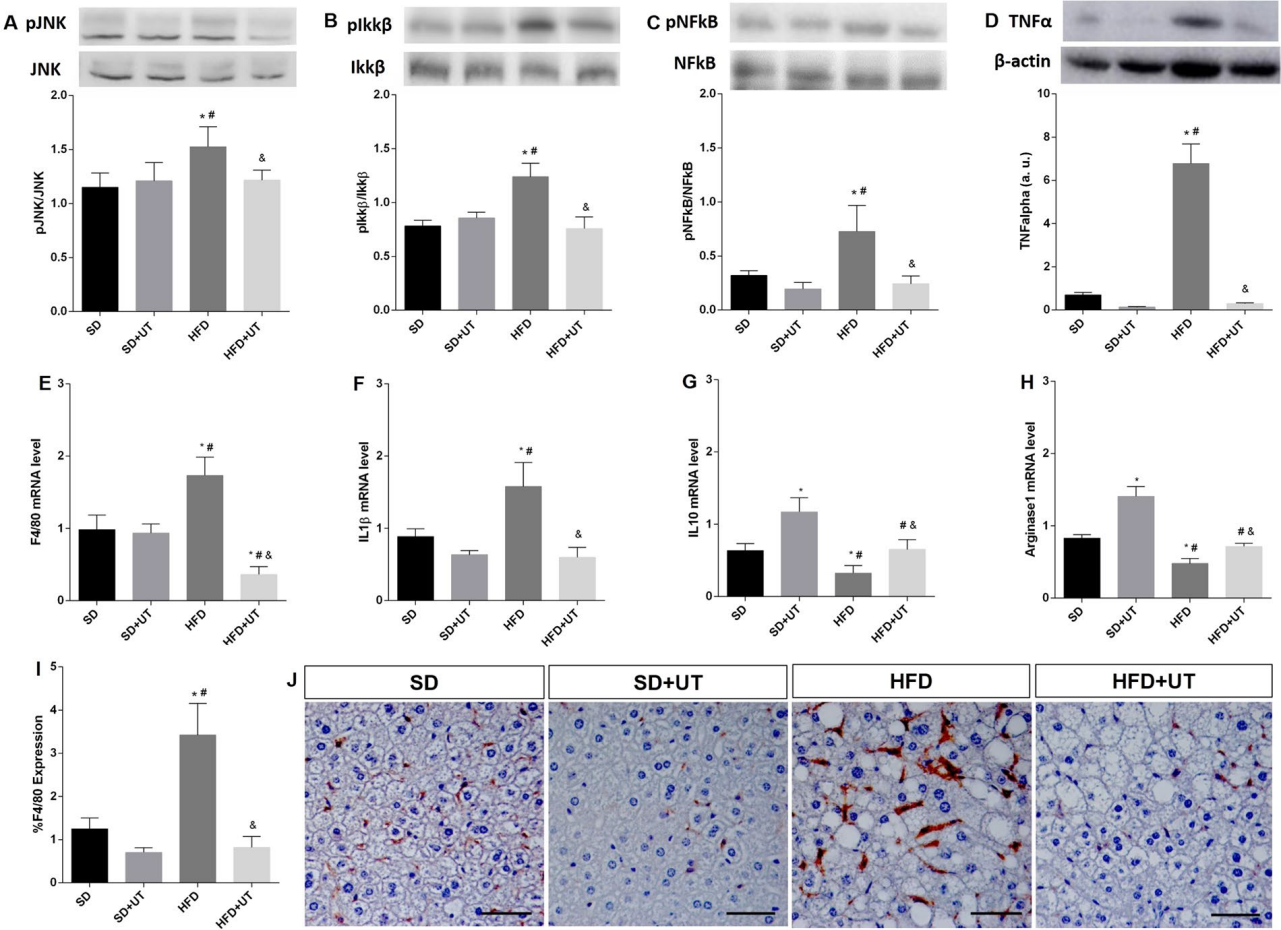
Effect of *Uncaria tomentosa* (UT) on the inflammatory signaling pathway in the liver of HFD mice. Liver samples of standard diet (SD) or high-fat diet (HFD) mice, treated with vehicle or the herbal extract (UT) were used for Western blot analysis (**A**–**D**) or for mRNA expression (**E**–**H**). Phosphorylated proteins were calculated by the ratio of the optical densitometry of phosphorylated and total protein expression. Protein expression data for other proteins was normalized to β-actin, n = 5. Photomicrographs of monocyte/macrophage marker F4/80 immunohistochemistry (brown color) in the liver of HFD mice model under treatment with UT, n = 4 (20× all photomicrographs, Scale bar = 50 μm) (**I**,**J**). The results are represented as mean ± SEM. The statistical differences as indicated by two-way ANOVA were as follows: *p < 0.05 (compared to SD), ^#^*p* < 0.05 (compared to SD+UT), ^&^*p* < 0.05 (compared to HFD), n = 5.

The JNK, Ikkβ, NFkB phosphorylation state were increased by 36%, 50% and 133%, respectively, (*p* < 0.05) in the HFD group when compared to the SD groups (vehicle:100% and UT: 100%). Furthermore, the UT treatment induced a reduction, in the HFD+UT group, of these intracellular proteins to values similar to those detected in the control groups (Fig. 7A–C).

TNF-α expression was 10-fold higher in the HFD group as compared to the SD group (6.8 ± 0.9 *vs*. 0.7 ± 0.1, *p* < 0.05). The UT treatment induced a substantial and significant reduction in the TNF-α expression levels, which approached a value similar to the control (0.3 ± 0.04, *p* < 0.05) (Fig. 7D).

In the HFD group, the mRNA expression of F4/80 and IL1β were increased by 170% and 177% (*p* < 0.05), respectively, when compared to the SD group (100%) (Fig. 7E,F). The UT treatment reduced the levels of F4/80 mRNA, in the HFD mice, to approximately 30% of the control detected and reverted the enhanced IL1β expression, observed in HFD group, to a value similar to that of the SD group. Concomitantly, the HFD reduced IL10 and arginase1 mRNA levels to 51% and 58% (*p* < 0.05), respectively, in HFD group compared to the SD group (100%) (Fig. 7G,H). However, there was a UT induced enhancement of IL10 and arginase 1 expression in the SD+UT group as well as a concomitant enhancement of these markers in the HFD+UT group to values similar to the SD group (p < 0.05).

The HFD group displayed an almost 3-fold increase (283%) in the number of F4/80-positive cells in the liver, and UT treatment reduced this to a value similar to that observed in the SD group (Fig. 7I,J).

**Figure 8:**
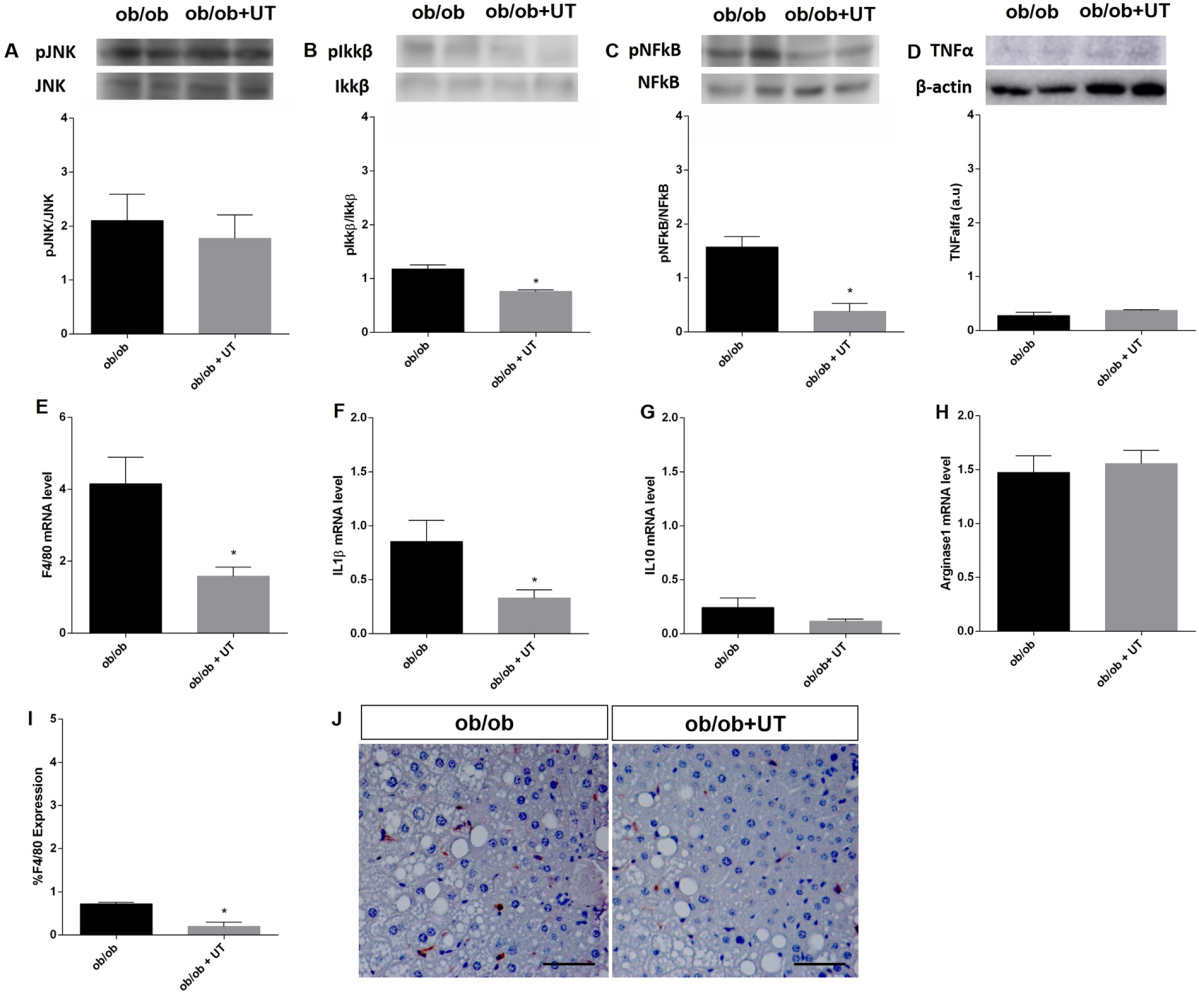
Effect of *Uncaria tomentosa* (UT) on the inflammatory signaling pathway in the liver of ob/ob mice. Samples of livers from ob/ob mice that received either vehicle (ob/ob) or UT extract (ob/ob+UT) were used for Western blot analysis (**A**–**D**) or for mRNA expression (**E**–**H**). Phosphorylated proteins were calculated by the ratio of the optical densitometry of phosphorylated and total protein expression. Protein expression data for other proteins was normalized to β-actin, n = 5. Photomicrographs of monocyte/macrophage marker F4/80 immunohistochemistry (brown color) in the liver of ob/ob mice under treatment with UT, n = 4 (20× all photomicrographs, Scale bar = 50 μm) **(I**,**J**). Data are presented as mean ± SEM, respectively. The statistical difference as indicated by Student’s *t*-test was, *p < 0.05.

The liver of the ob/ob+UT group had reduced levels of phosphorylated Ikkβ and NFkB compared to the ob/ob vehicle mice, to 80%, and 20%, respectively, p < 0.05 (Fig. 8B,C). However, treatment did not affect TNF-α expression in these obese mice (Fig. 8D).

Furthermore, the UT treatment induced a reduction to approximately 30% of the values found in saline treated mice of the F4/80 and IL1β mRNA levels (Fig. 8E,F). There was no difference between the anti-inflammatory markers IL-10 and Arginase-1 mRNA levels as compared to the respective control group (Fig. 8G,H). The F4/80-positive cells in the liver of UT treated ob/ob mice were reduced to approximately 30% of the values found in saline treated mice (Fig. 8I,J).

The redox state analyzed through lipid peroxidation (TBARS), nitrotyrosine and carbonylation detection in liver samples showed no statistical significance among groups, with the exception of a reduction in the carbonylation levels observed in the ob/ob+UT group, when compared to the ob/ob vehicle group (Supplementary Information).

Overall, our results indicate that administering UT to obese mice induced an improvement in the anti-inflammatory state of the liver and may be considered a potential therapeutic intervention for enhancing hepatic insulin sensitivity and in the reversion of NASH to a less harmful NAFLD.

## Discussion

We investigated the effect of a herbal extract from the *Uncaria tomentosa* plant on insulin sensitivity, NAFLD features, energy expenditure, and inflammatory markers in the liver in two obese rodent models: the high fat diet (HFD) and the genetically obese (ob/ob) mice. In the HFD model, UT treatment reduced the BMI by 20%, enhanced energy expenditure, improved the insulin sensitivity, and reduced the features associated with NASH. Furthermore, in the ob/ob mice, there was an improved glucose tolerance with an enhanced insulin blood level during fasting.

It is interesting that UT treatment improved insulin sensitivity in the HFD fed mice, with no change in glucose tolerance. However, the HFD+UT group displayed reduced fasting plasma insulin, which suggests improved insulin sensitivity as observed with the insulin tolerance test. Also, it suggests that plasma insulin would be reduced over the course of the glucose tolerance test, with no change in plasma glucose, characterizing improved insulin sensitivity. In the ob/ob mice, UT treatment was not able to overcome the severe insulin resistance observed in this mouse model, since this is a type 2 diabetes (T2D) rodent model, which display a much more severe insulin resistance compared with rodents fed a HFD. Moreover, T2D rodent models and humans usually display impaired glucose-stimulated insulin secretion. Interesting, UT treatment enhanced fasting plasma insulin levels in ob/ob mice, probably due to improved glucose-stimulated insulin secretion, suggesting that plasma insulin would be increased in UT-treated mice over the course of the glucose tolerance test, consequently, improving glucose tolerance in these mice.

It has been widely reported that a chronic low inflammatory condition is associated with obesity^15,16^. The hypertrophic adipocytes exhibit impaired lipolysis and lipid esterification activities that lead to an increase in plasma fatty acid (FA) levels, a reduction in FA buffering capacity, and prompt accumulation of ectopic fat^17^, which has been widely associated with hepatic insulin resistance^18–20^_._

Saturated fatty acids can bind to the toll like receptor type 4 (TLR-4) and activate inflammatory responses in macrophages, dendritic cells, adipose tissue and liver. The TLR-4 activates the Ikkβ/NFĸB and MAPK (p38, JNKs) intracellular signaling pathway, which in turn leads to the transcription of pro-inflammatory cytokines^21–23^. Ikkβ and JNK are also able to induce serine phosphorylation of IRS-1, which impairs the insulin receptor signaling, and leads to insulin resistance^17,24–27^. The reduced levels JNK, Ikkβ, NFkB and TNF-α in the UT treated mice reinforces the fact that this extract possesses anti-inflammatory properties. Furthermore, ob/ob+UT displayed lower levels of carbonylation, which may indicate higher ROS production in this hypoleptinemic mouse compared to HFD feeding used herein. Thus, this data amplifies the potential anti-oxidant effect of *Uncaria tomentosa* extract.

In agreement with previous studies, the liver insulin resistance caused by lipid accumulation was associated with increased IRS-1 serine phosphorylation^27–31^. The reduction of IRS-1 serine phosphorylation induced by the UT treatment is likely related to an improvement in insulin sensitivity in the liver of both the HFD and ob/ob mice. Taking together the anti-inflammatory effect of the UT and the reduced IRS-1 serine phosphorylation, it is plausible that the enhanced downstream insulin receptor signaling was due to enhanced substrate phosphorylation and consequently intracellular signal transduction, rather than an enhanced IR kinase activity itself.

Features associated with steatohepatitis include: intracellular lipid droplets, inflammatory cellular infiltrates, collagen fibrils, increased mRNA levels of IL-1 and F4/80, F4/80-positive cells in the liver and higher blood ALT activities. In the HFD mice all seven of these features were detected, while in the ob/ob mice only five of these features were observed. In this regard, both obese mice models have a pattern of NAFLD with active inflammatory infiltrate. However, the HFD mice display a more harmful condition, while the ob/ob mice, despite having a higher BMI, showed a less harmful type of fatty liver disease. Despite the discrepancy in the intensity of the NAFLD, UT treatment reduced the intracellular fat accumulation and liver inflammatory markers in both obese mouse models. It is also worth mentioning that the SD+UT group displayed no alteration in plasma ALT and AST activities, which indicates that there is no cytotoxic liver effect. However, the lack of ALT blood activity reduction in the HFD+UT mice might be related to discrepancies between the short term period of treatment and the metabolic clearance of the enzyme in the blood stream. In fact, five days of treatment corresponds to almost 0.7% of the 24 month of lifespan of a normal mouse, while the recommended UT treatment in humans should last about 0.6% of his/her 80 years life expectancy.

Despite the protective and potentially reversible effect of UT on the NAFLD in both obese models, the treatment was more effective in the HFD obese mice than in the ob/ob mice. The distinct features related to the mechanism behind the two obese animal models may play a role in the response to the UT treatment against the NAFLD co-morbity: (1) absence of functional leptin versus leptin resistance, (2) hypogonadism due to persistent immaturity of the hypothalamus-pituitary gonadal axis in the ob/ob mice, (3) the degree of sarcopenia associated with obesity and insulin sensitivity impairment, and (4) the degree of chronic inflammation. However, we do not know if prolonged UT treatment could interfere with some of the other differences, besides the inflammatory status and leptin sensitivity, observed between these obese animals.

There is evidence that indicates that both patterns of steatosis, the presence of macrovesicles or microvesicules, are associated with an impairment in mitochondrial function^32^. Furthermore, weight gain and weight loss are associated with changes in energy expenditure. Most of these changes are non-adaptive and occur passively, but can also be adaptive and arise actively. In fact, the adaptive thermogenesis (AT) consists of (1) resting energy expenditure and the diet-induced thermogenesis, and (2) activity-related energy expenditure. The mechanism behind the AT is considered to be under hormonal and genetic control, particularly due to insulin, leptin, thyroid hormones and sympathetic nervous system activity^33^.

In this regard, the reduced energy expenditure and increased BMI detected in the obese mice may be related to both insulin resistance and impaired leptin action. Despite the similar amount of blood leptin levels observed in fasted HFD mice, one possible cellular mechanism by which the UT treatment improved the energy expenditure associated with reduced BMI could be through an increase in hypothalamic leptin sensitivity in the HFD mice. The fact that UT is unable to modulate BMI and energy expenditure in the ob/ob mice supports this proposition, since the main feature of this obesity model relies on the absence of functional circulating leptin^34^.

In addition to already being clinically used in patients with arthritis^10^, probably due to its anti-inflammatory effects, *Uncaria tomentosa* extract treatment improved insulin sensitivity, attenuated liver inflammation and hepatic fat accumulation in both HFD fed mice and ob/ob mice. Taken together, these data provide evidence, for the first time to our knowledge, that *Uncaria tomentosa* treatment may have a potential utility as a therapeutic approach for obesity and NASH-associated metabolic disease.

## Material and Methods

### Animals

Male C57BL/6 mice, weighing 20–25 g, were obtained from the animal facility at the University of São Paulo (USP) Medical School and were maintained in a temperature controlled room at 22 ± 2 °C with free access to food and tap water, and light-dark cycle of 12 hours (light on from 6 am to 6 pm). The ob/ob (genetically obese) mice were obtained from the Department of Physiology and Biophysics, Institute of Biomedical Sciences – ICB-USP, weighing 55–60 g, and were maintained under the same conditions as described above. There were initially 8 animals per group and the statistical analyses performed considered at least 5 animals per group. Standard Diet (SD) consisted of commercial rodent chow (Nuvilab® - CR-1, Curitiba, Parana, Brazil) containing 22% protein, 61% carbohydrate, 4.5% fat, 8% cellulose and 4.5% vitamins and minerals, which provided 4.5 kcal/g of chow. The high fat pelletized diet (HFD) contained 20.5% protein, 41% carbohydrate, 34% fat and 4.5% vitamins and minerals, which provided 6.1 kcal/g of chow (PragSoluçoes Biosciences, Jau, Sao Paulo, Brazil). All experimental procedures were performed following the “Guidelines for the ethical use of animals in applied etiology studies”^35^ and were previously approved by the Ethics Committee on the use of animals at the ICB-USP (Protocol: No. 035, in page 30, book 03).

### Treatment

#### Extract composition

The crude extract of *Uncaria tomentosa* was donated from Herbarium Laboratorio Botanico (herbarium.com.br) (PR, Brazil). The chromatogram of the extract sent by the supplier confirms the presence of uncarine D, uncarine F, mitraphylline, isomitraphylline, uncarine C and uncarine E (Supplementary Information).

A preliminary dose response test using 50, 100 and 200 mg.kg^−1^ b.w. after 1, 5 and 10 consecutive days was performed (data not shown). It was determined that the smaller effective dose (50 mg.kg^−1^) administered for 5 consecutive days improved insulin sensitivity, thus all the experiments were performed following the regimen of: 50 mg.kg^−1^ b.w., once a day, for 5 consecutive days.

The insulin resistant animals (HFD and ob/ob) received *Uncaria tomentosa* crude extract diluted in saline through gavage once a day for 5 consecutive days, at maximum dose volume of 200 μL each time. The used daily dose of 50 mg.kg^−1^ b.w. is equivalent to a human dose of 1 tablet (100 mg), 3 times a day. The dosage used with the human dose recommendation was calculated according to Reagan-Shaw *et al*.^36^.

### Insulin tolerance test (ITT) and Glucose tolerance test (GTT)

The insulin tolerance (ITT) and glucose tolerance (GTT) tests were performed in the afternoon after 6 h of fasting^37,38^. Blood samples were collected from the tail vein for basal blood glucose level measurements. For the ITT, awake mice received intraperitoneal injection of regular insulin diluted in saline solution (0.75 mU.g^−1^ and 3 U.kg^−1^ of body weight, HFD and ob/ob, respectively). Tail blood samples were collected at 15, 30, 60 and 90 minutes after the intraperitoneal insulin injection^39^. For the GTT, the animals received a solution containing glucose (1 mg.g^−1^ b.w.)^40^, and aliquots of blood were collected at 15, 30, 60, 90, and 120 minutes after the glucose infusion. The blood was applied to reactive strips (Roche®, São Paulo, Brazil) and glucose levels were measured using an Accu-chek Active apparatus (Roche, Mannheim, Germany).

### Food intake, energy expenditure and body mass index

To determine food intake an animal monitoring system (CLAMS) was employed (Columbus Instruments, USA) and energy expenditure was monitored through oxygen (VO_2_) consumption and carbon dioxide (VCO_2_) production. The animals remained in the system for 7 days, 2 days of acclimatization period and 5 days receiving either saline as vehicle or crude extract of UT diluted in saline, and the results were analyzed during the last 3 days of this period. At the end of the experimental period, the body weight (g) and the naso-anal length (cm) were measured to calculate the body mass index (BMI = g.cm^−2^).

### Liver Morphology

#### Hematoxylin/Eosin, Picrocirius, and Masson Trichrome staining

To evaluate liver morphology, the liver was fixed in 10% formaldehyde solution for 8 hours, in individual cassettes. Subsequently, the fixed samples were kept overnight in 70% alcohol. The samples were then dehydrated through a series of baths in 95% alcohol, 100% alcohol and xylene. Once the samples were dehydrated, the tissue samples were embedded in paraffin at 60 °C. A microtome (Zeiss, Jena, Germany) was used to cut the samples into 5 micron slices. The slices were stained with hematoxylin and eosin (H&E), and the cellular morphology was evaluated and the intracellular vacuoles were quantified. Twenty images from each animal were obtained using a Nikon Eclipse Ti-U microscope at 20× magnification coupled with a Nikon DS-R1 digital camera and NIS-Elements BR 3.1 software. These images were projected onto a high resolution LCD monitor. The test system produced from a transparent sheet was placed on the monitor screen, this system contained 270 points symmetrically distributed through the sheet and the total number of points were named as P (P = 270). Each point that remained on the fat droplets was counted and named as IP. Then the IP/P ratio was calculated and the number derived from it corresponded to the amount of fat droplets^4^. The results were expressed as a percentage.

The slices were also submitted to Picrosirius and Masson trichrome staining to identify collagen fibers. Twenty-five images using 20x magnification (3 distinct slices) from 3 animals per group were analyzed. The abundance of collagen deposition in the perivenular areas of the liver was presented as the mean of the percentage of the total portal spaces from each animal per group.

#### Oil Red O (ORO) Staining

The tissue-tek initially embedded liver samples (Thermo Scientific, Massachusetts, USA) were placed in isopropanol alcohol and immediately frozen in liquid nitrogen (N_2_). Twelve micron slices were prepared using a cryostat (Microm H560 Thermo Scientific, Massachusetts, USA). Three slices from different parts of the samples were disposed per slide, and two slides per animal were used. The slides were stained with ORO and Mayer’s hematoxylin. Ten images from each animal were obtained using a microscope with 20× objective magnification. The identification of ORO stained area was performed using the ImageJ program^41^.

#### Immunohistochemistry (IHC)

For immunohistochemistry studies the liver was fixed in a 10% formaldehyde solution for 8 hours. After the samples were dehydrated, the tissue samples were embedded in paraffin at 60 °C. Five micron thick tissue sections were cut transversely on a microtome (Zeiss, Jena, Germany) and antigen retrieval was performed by heating (96 °C) the specimen sections in sodium citrate buffer. The sections were washed with PB, incubated with 3% hydrogen peroxide to inhibit endogenous peroxide and treated with 10% goat serum for 1 hour at 22 °C in a humidified chamber. Next, all the sections were then incubated overnight at 4 °C with the primary rat polyclonal antibody F4-80 (Bio-Rad AbD Serotec, UK) diluted in PBS (1:200). The sections were then incubated with the appropriate secondary antibody (Sigma-Aldrich, USA) for 1 hour at RT. For the visualization of immune complexes the vectastin ABC-kit (Vector Laboratories, CA, USA) was applied for 1 hour at RT for signal amplification and 3,3-diaminobenzidine diluted in PBS containing 0.03% (v/v) H_2_O_2_ was utilized as the chromogen, yielding the overall brown color. Negative controls were conducted for each antibody by omitting the primary antibody. At least two samples from each animal were independently analyzed by two experienced investigators. Images were acquired with a Nikon DS-R1 digital camera connected to a Nikon Eclipse Ti-U microscope. The immunostained sections were quantified by using the Image Pro Plus software (Media Cybernetics, MD, USA). At least 5 areas per slide were selected and photographed in a blinded manner. The expression of F4/80 was calculated as follows: the total area of liver (specimen) was outlined, and the area occupied by cells expressing F4/80 were quantified using the image analyser within the reference area. A color for F4/80 was pre-defined and applied to the selected area. The result was expressed as a percentage of positive area in relation to the total area.

### Western blot analysis

Prior to removing the liver for western blot analysis, animals were deeply anaesthetized with thiopental (50 mg/kg b.w.), and after loss of corneal reflexes the abdominal wall was opened to visualize the liver and portal vein. In order to analyze the early steps of intracellular insulin action, some mice received a bolus saline infusion through the portal vein with or without regular insulin (1U) (Humulin R, Eli Lilly of Brasil, SP, Brazil). Thirty seconds after the insulin injection or euthanasia, the liver was removed and homogenized in lysis buffer (100 mM Tris, 10% SDS, 100 mM sodium pyrophosphate, 100 mM sodium fluoride, 10 mM EDTA, 10 mM sodium orthovanadate, pH 7.4). The tissue extracts were centrifuged at 15,294 × g, at 4 °C, for 40 minutes. The protein content of the supernatants was measured by the Bradford method. Aliquots of the supernatant, containing 50 ug total protein, were treated with Laemmli buffer supplemented with 200 mM dithiothreitol, loaded onto 8% and 10% gels and subjected to SDS-PAGE. The proteins were then transferred from the gels to nitrocellulose membranes using a Bio-Rad mini trans blot Apparatus (USA). The membranes were incubated in TBST-B blocking buffer (10 mM Tris, 150 mM NaCl, 0.05% tween 20, and 5% skim milk) for 2 hours at RT, to prevent any nonspecific binding to the membrane. The nitrocellulose membranes were incubated with the specific primary antibodies overnight at 4 °C, and subsequently incubated with secondary antibody conjugated to horseradish peroxidase for 1 hour, at RT. The immunoblots were developed using the SuperSignal® West Pico Chemiluminescent Substrate (Thermo Scientific, USA). Visualization of the immunoblots was performed using an Amersham^TM^ Imager 600 and quantified using the ImageJ software (imagej.net/Downloads). The primary antibodies used were: IR (catalog number 3025S), p-insulin receptor B (catalog number 3026 s), pIRS1 (catalog number 2385 s), pNFkB (catalog number 3031 S), NFkB (catalog number 8242 S), pIkkβ (catalog number 5176) and TNF-α (catalog number 3707 s), Cell Signaling, Massachusetts, USA; pJNK (catalog number 6254), pAKT (catalog number 7985-R) and β-actin (catalog number 130657) were from Santa Cruz, Dalas, USA; IRS-1 (catalog number 06-248) and pSer^307^-IRS-1 (catalog number 07-247) were from Upstate, New York, USA; AKT (catalog number 126811), Abcam, San Francisco, USA; JNK (catalog number 252355), and Ikkβ (catalog number 725818) were from R&D Systems, Minneapolis, USA.

### Real time polymerase chain reaction (RT-PCR)

Total RNA from the liver was extracted with Trizol reagent (Thermo Scientific, Massachusetts, USA) and reverse transcribed into cDNA (High-Capacity cDNA kit, Applied Biosystems, USA). Gene expression was evaluated by RT-PCR using a Rotor Gene Q (Qiagen, USA) and SYBR Green as the fluorescent dye (Platinum® SYBR® Green qPCR Supermix UDG, Invitrogen, USA).

The primer sequences were: IL1β Forward: 5′-GGCAGCTACCTGTGTCTTTCCC-3′ Reverse: 5′-ATATGGGTCCGACAGCACGAG-3′; IL10 Forward: 5′-TGCCAAGCCTTATCGGAAATG-3′ Reverse: 5′-AAATCGATGAGAGCGCCTCAG-3′; F4/80 Forward: 5′-CCTGAACATGCAACCTGCCAC-3′ Reverse: 5′-GGGCATGAGCAGCTGTAGGATC-3′; Arginase 1 Forward: 5′-GGAAGAGTCAGTGTGGTGGTGC-3′ Reverse: 5′-CAGGAGAAAGGACACAGGTTGC-3′. The gene B2M Forward: 5′-CCCCACTGAGACTGATACATACG-3′ Reverse: 5′-CGATCCCAGTAGACGGTCTTG-3′ was used as the constitutive gene. The gene expression analysis was carried out using a method previously described by Livak and Schmittgen (2001)^42^ and Pfaffl (2001)^43^.

### Statistical analysis

The results were analyzed using the GraphPad Prism version 6.0® program (GraphPad Software, La Jolla, CA, USA). The minimum sample size per group for each parameter analyzed was defined by a *n* sufficient to perform the analysis of distribution of samples through the “D′Agostino and Pearson omnibus normality test” recommended by the GraphPad Prism version 6.0® program. All samples were evaluated for normal distribution and subjected to either a two-way ANOVA followed by the post-hoc Bonferroni test (Bonferroni Multiple Comparison Test) or a *Student-t* test (p < 0.05). The results were expressed as mean ± standard error of mean (mean ± SEM).

## Electronic supplementary material

Supplemental Information
