# Supplementary material for: Uncaria tomentosa improves insulin sensitivity and inflammation in experimental NAFLD
Source: Sci Rep. 2018 Jul 20;8:11013. doi: 10.1038/s41598-018-29044-y (PMC6054645; doi:10.1038/s41598-018-29044-y)
Supplement: Supplementary file 1 — Supplemental Information [file 41598_2018_29044_MOESM1_ESM.docx]

***Uncaria tomentosa* improves insulin sensitivity and inflammation in experimental NAFLD**

**Layanne CC Araujo^1^, Karla B Feitosa^2^, Gilson M Murata^1^, Isadora C Furigo^1^, Simone A Teixeira^2^, Camila F Lucena^1^, Luciene M Ribeiro^1^, Marcelo N Muscará^2^, Soraia KP Costa^2^, José Donato Jr^1^, Silvana Bordin^1^, Rui Curi^3^, Carla RO Carvalho^1*^**

^1^ Department of Physiology and Biophysics, ^2^ Department of Pharmacology, Institute of Biological Science, University of São Paulo, São Paulo, 05508-900, **^3^**Interdisciplinar Post-Graduate Program in Health Sciences, Cruzeiro do Sul University, São Paulo, SP, Brazil.

*Corresponding author: croc@icb.usp.br

**Supplementary Information:**


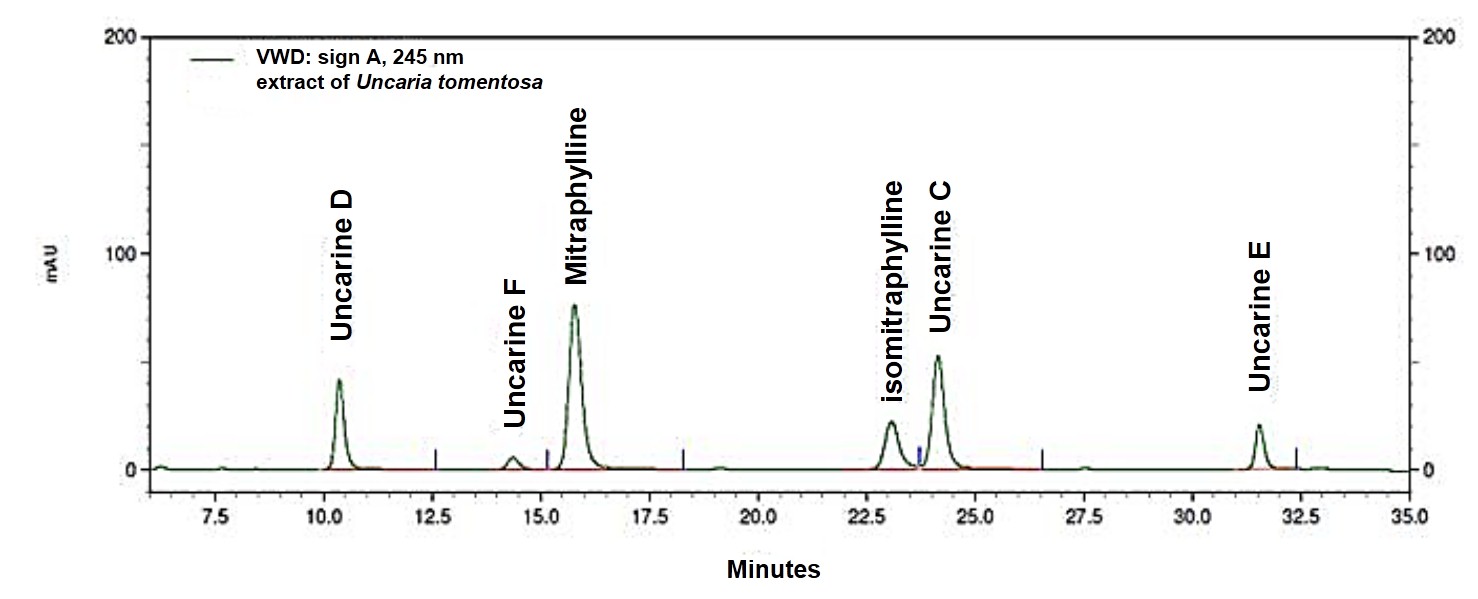


**SI 1:** Chromatogram of the crude extract of *Uncaria tomentosa* used in the experiments, provided by the pharmaceutical supplier, Herbarium. The crude extract of *Uncaria tomentosa* contains alkaloids such as uncarine D, uncarine F, mitraphylline, isomitraphylline, uncarine C, uncarine E, as well as mitraphylline (5.97%), which is a major component of the alkaloids (La Paz et al., 2016).


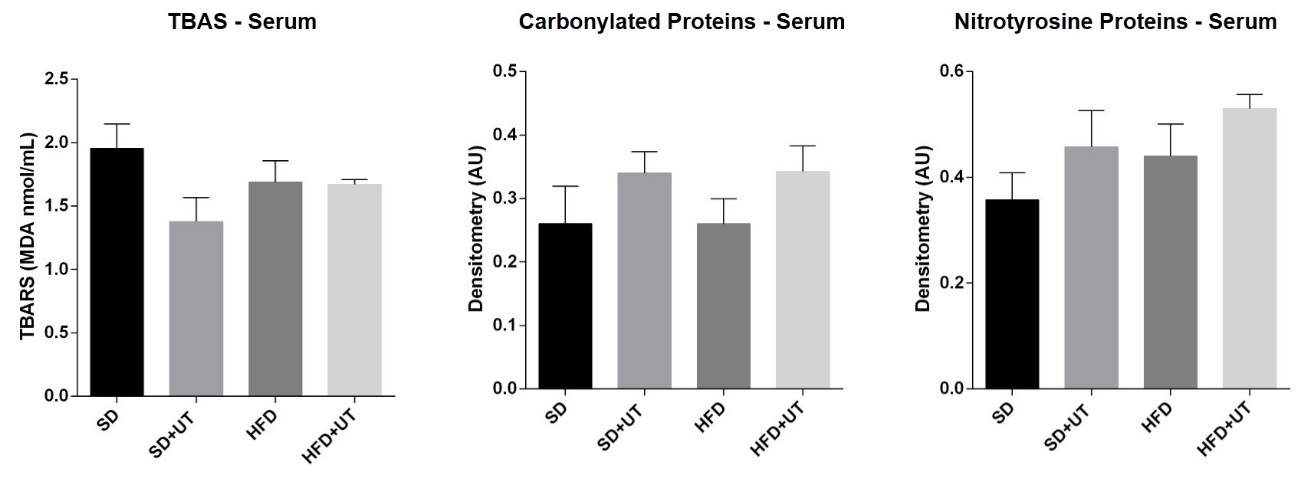

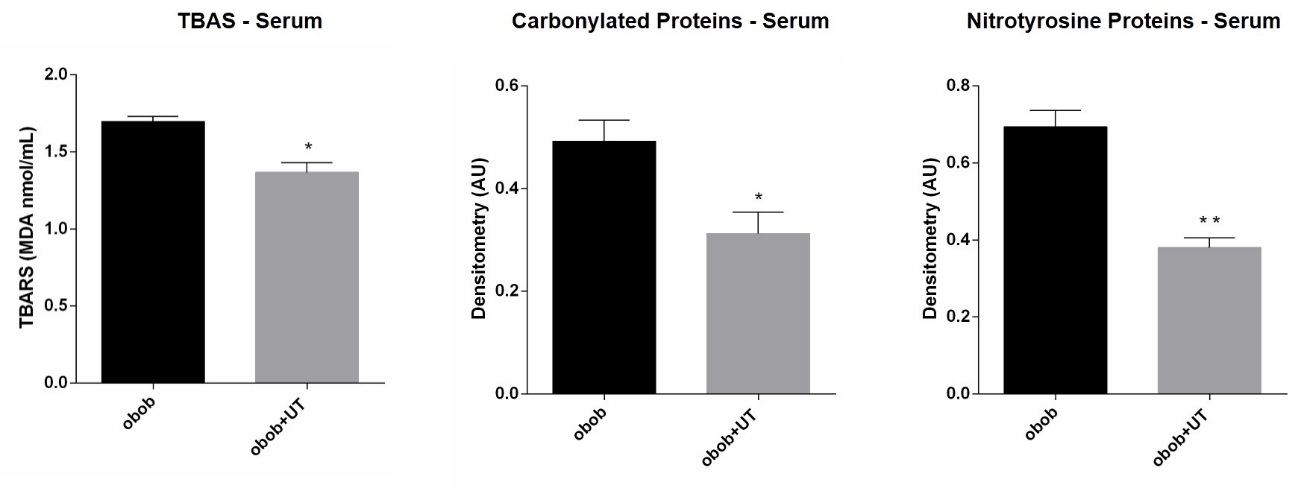


**A.**

**B.**


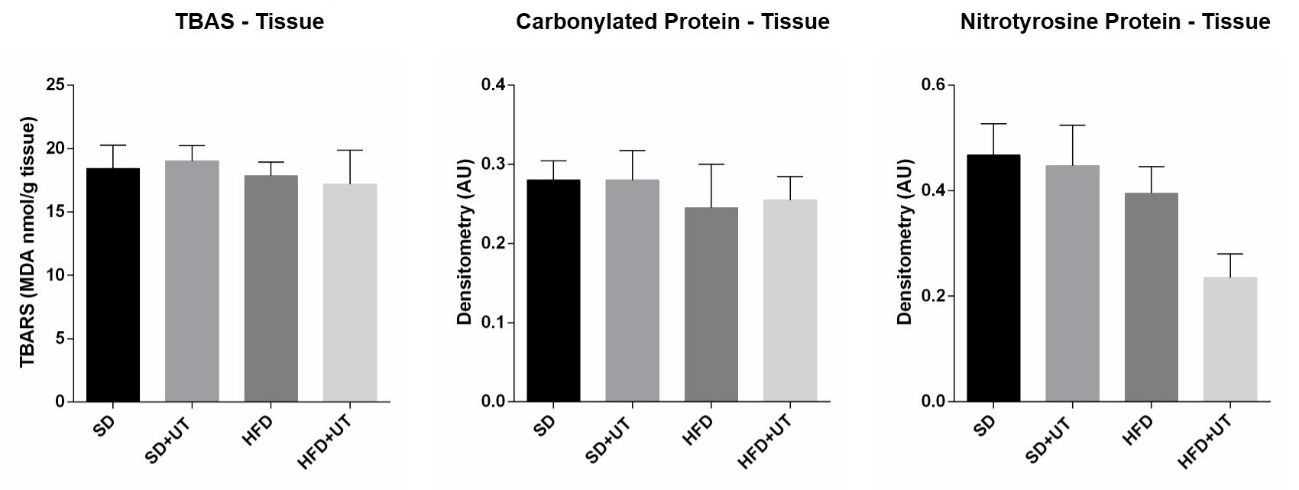

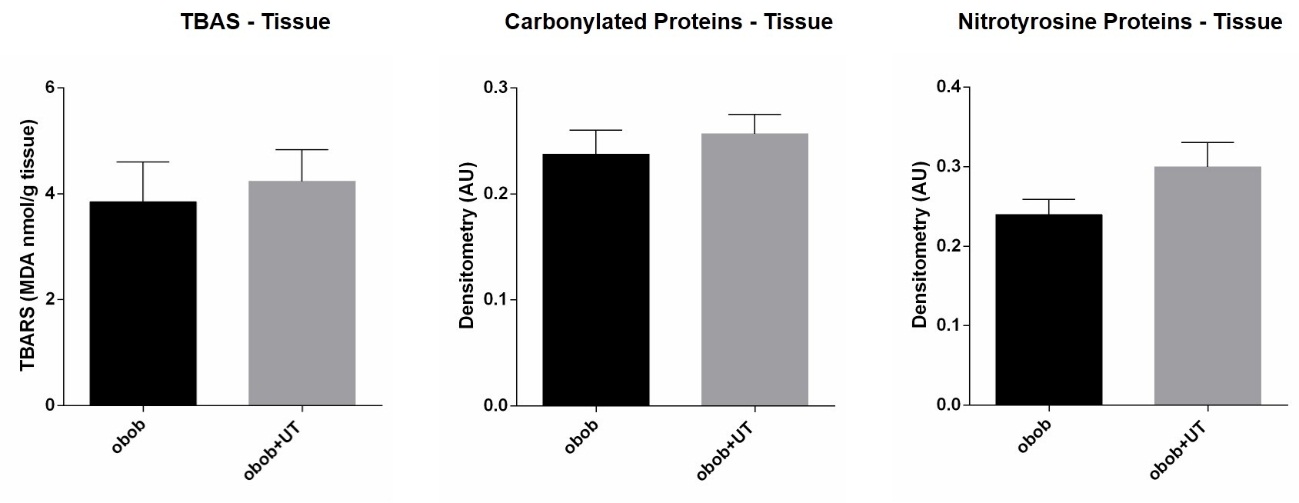


**C.**

**D.**

**SI 2:** ROS analyses in obese and lean animals.

After 5 days of vehicle or *Uncaria tomentosa* (UT) crude extract treatment lean, HFD-induced obese, and genetically obese ob/ob mice were euthanized. ROS were analyzed using the TBARS assay and through the detection of carbonylation and nitrotyrosine in both the serum (A,B) and liver fragments (C,D) of mice. The values in the bar graphs bars represent the mean ± SEM. The statistical analyses were performed using a two-way ANOVA (HFD versus SD) or with the Student’s t-test (ob/ob). **p* < 0.05, ***p* < 0.01 n = 4-5.
